# A PAGE screening approach for identifying CRISPR-Cas9-induced mutations in zebrafi sh

**DOI:** 10.2144/btn-2018-0012

**Published:** 2018-06

**Authors:** Ariel J VanLeuven, Sungdae Park, Douglas B Menke, James D Lauderdale

**Affiliations:** 1 Department of Cellular Biology, University of Georgia, 724 Biological Sciences Building, Athens, GA 30602-2607, USA; 2 Department of Genetics, University of Georgia, Paul D Coverdell Center, 500 D.W. Brooks Drive, Room 250, Athens, GA 30602, USA; 3 Faculty in the Neuroscience Division of the Biomedical & Health Sciences Institute (BHSI), University of Georgia, Paul D Coverdell Center, 500 D.W. Brooks Drive, Athens, GA 30602-7394, USA

**Keywords:** *Danio rerio*, genotyping, polymerase chain reaction (PCR)

## Abstract

The introduction of CRISPR-Cas9 technology for targeted mutagenesis has revolutionized reverse genetics and made genome editing a realistic option in many model organisms. One of the difficulties with this technique is screening for mutations in large numbers of samples. Many screening approaches for identifying CRISPR-Cas9 mutants have been published; however, in practice these methods are time consuming, expensive, or often yield false positives. This report describes a PCR-based screening approach using non-denaturing PAGE. This approach does not depend on the formation of heteroduplexes and reliably detects changes as small as 1 base-pair (bp) in nucleic acid length at the target site. This approach can be used to identify novel mutations and is also useful as a routine genotyping method.

## METHOD SUMMARY

Our approach implements a PAGE technique that is known to provide resolution of as small as 1 bp in 1000 bp as an inexpensive and robust screening approach for identifying CRISPR-Cas9-induced mutations in zebrafish. In this approach, we PCR amplify a small region (<150 bp) encompassing the CRISPR-Cas9 target site and the PCR product is then directly run on a 10 × 8 cm, 15% polyacrylamide gel at 200 Volts for 2–2.5 h. Using this approach, we routinely detect 1–14 bp indels without relying on the formation of heteroduplexes prior to PAGE.

Our approach implements a PAGE technique that is known to provide resolution of as small as 1 bp in 1000 bp [1, 2] as an inexpensive and robust screening approach for identifying CRISPR-Cas9-induced mutations in zebrafish. The CRISPR-Cas9 genome editing technique is widely used in many labs, especially in the zebrafish community [3–5]. In our experience, the rate-limiting step when using this technology is the screening of zebrafish for CRISPR-Cas9-induced mutations. Several techniques describing the identification of CRISPR-Cas9-induced mutations have been reported, each with their own strengths and limitations [6–14]. T7 Endonuclease I (T7E1) and Surveyor Mismatch Cleavage Assays, both PCR- and molecular-based assays, are efficient in identifying mismatched DNA at a specific locus; however, these assays also detect single-nucleotide polymorphisms (SNPs). SNPs are prevalent in the zebrafish genome, and in our hands, use of the T7E1 assay leads to false-positive results for our genes of interest. High-resolution melting analysis (HRMA) and derivative melting curves require a quantitative PCR machine that can be expensive to implement if the equipment and software are not already in a laboratory. Furthermore, the derivative melting curve assay is best used to detect mutations that have a change of greater than 15 bp in nucleic acid length at the target site [10]; however, the median CRISPR-Cas9-induced indel size ranges from 4–9 bp depending on the length of the single-stranded guide RNA (sgRNA) [15]. Sequencing is definitive in identifying indels of any size but can be expensive and slow for a primary screening approach.

In our laboratory, CRISPR-Cas9 is used as a tool to create and establish mutants for specific genes of interest in zebrafish. To facilitate screening, we tested neutral PAGE as a rapid and sensitive method for identifying CRISPR-Cas9 mutants. There are assays that use PAGE to identify CRISPR-Cas9-induced mutations in zebrafish, mice and human cells; however, these assays require heteroduplex formation prior to PAGE [13,1 4]. We reasoned that it would be possible to directly run PCR products via PAGE both to identify new mutations and to genotype zebrafish with known mutations based solely upon a size difference in amplicon length rather than through formation and detection of heteroduplexes or enzymatic cleavage of DNA mismatches. The detection of small changes in nucleotide length, such as those of a typical CRISPR-Cas9 indel, requires a high-percent polyacrylamide gel. We use gels containing a 15% concentration of acrylamide monomer to obtain sufficient resolving power in amplicons ranging from 25–150 bp [2]. Importantly, we find that an acrylamide monomer to N,N′-methylenebisacrylamide crosslinker ratio of 19:1 (or 5%) is essential to resolve 1–2-bp indels (supplemental protocol).

**Figure 1.**
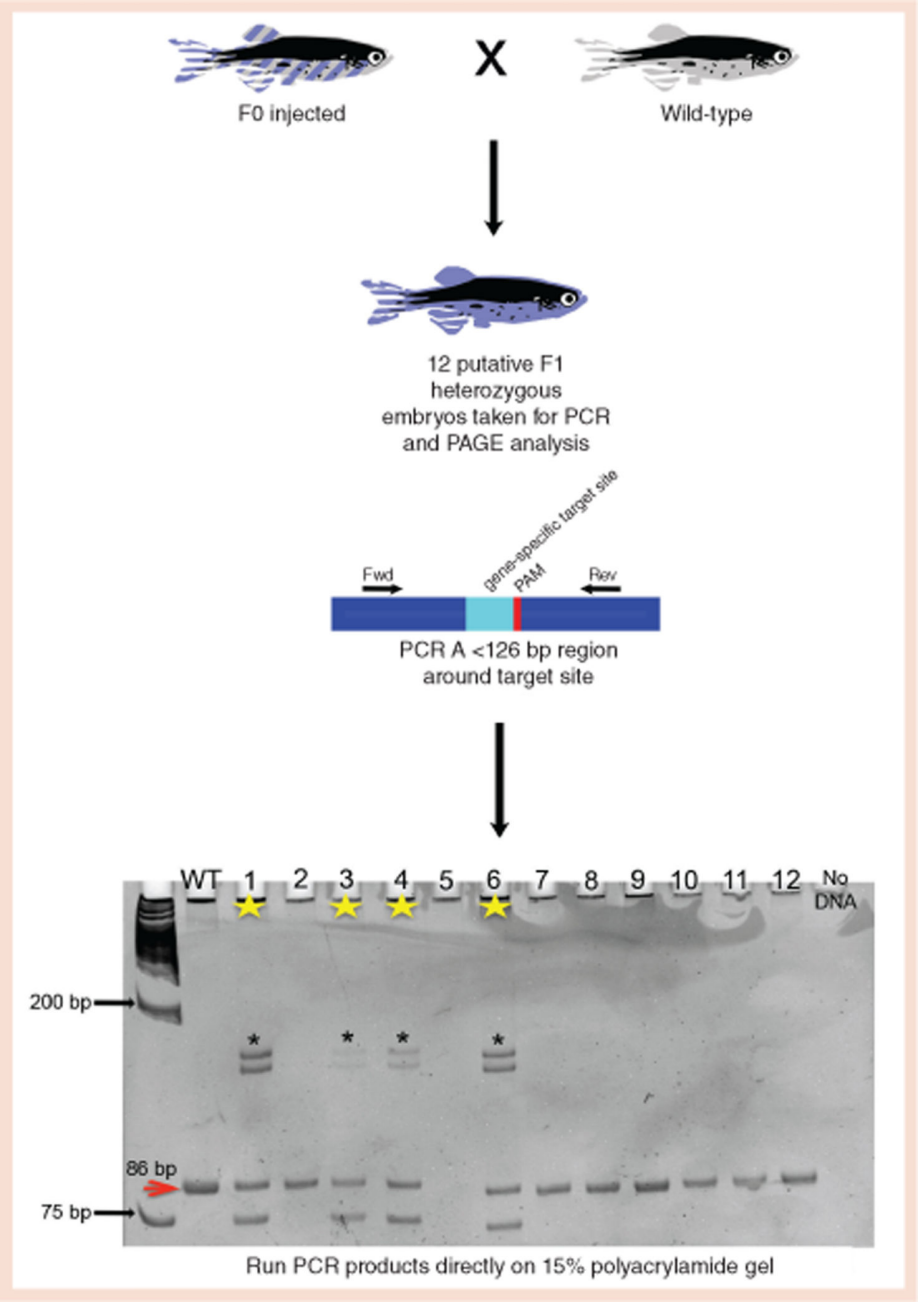
Workflow overview of PCR and PAGE for screening CRISPR-Cas9-induced mutations in zebrafish. F0-injected zebrafish are grown to adulthood and outcrossed to a wild-type zebrafish. 12 embryos from this outcross are sacrificed for genomic DNA extraction and PCR analysis of the region encompassing the target site. PCR products are directly run on a 15% polyacrylamide gel. This gel represents an outcross in which the F0-injected founder is carrying a single 10-bp deletion at *gad2* exon 1 that is transmittable at a frequency of ∼33% to the F1 generation. The second pair of bands that are noted with an asterisk are heteroduplexes. These heteroduplexes are seen with all heterozygous samples for all alleles and are not a reflection of nonspecific primer binding.

In a typical screening experiment, CRISPR-Cas9-injected embryos, referred to as F0 injected, are grown to sexual maturity and then outcrossed to a wild-type zebrafish to obtain putative F1 heterozygous progeny. Because a single founder could harbor many germline mutations, we screen zebrafish individually at 2 days post-fertilization (dpf) via PCR and PAGE analyses. If an animal has a CRISPR-Cas9-induced mutation at the target site, there will be two bands on the gel: one band of known size that represents the wild-type allele and an additional band that represents a CRISPR-Cas9-induced indel (Figure 1).

To perform these experiments, we select 12 zebrafish per F0 outcross, and then perform a standard DNA extraction and ethanol precipitation on individual embryos. We use ∼100 ng of genomic DNA for a standard PCR reaction with gene-specific primers. We directly load 5 μl of the PCR product into a 10 × 8 cm, 15% polyacrylamide gel and run the gel at 200 V for 2–2.5 h in freshly prepared 1X TBE buffer. The gel is stained with ethidium bromide and analyzed under UV light. A detailed supplemental protocol is also available for this assay.

**Figure 2.**
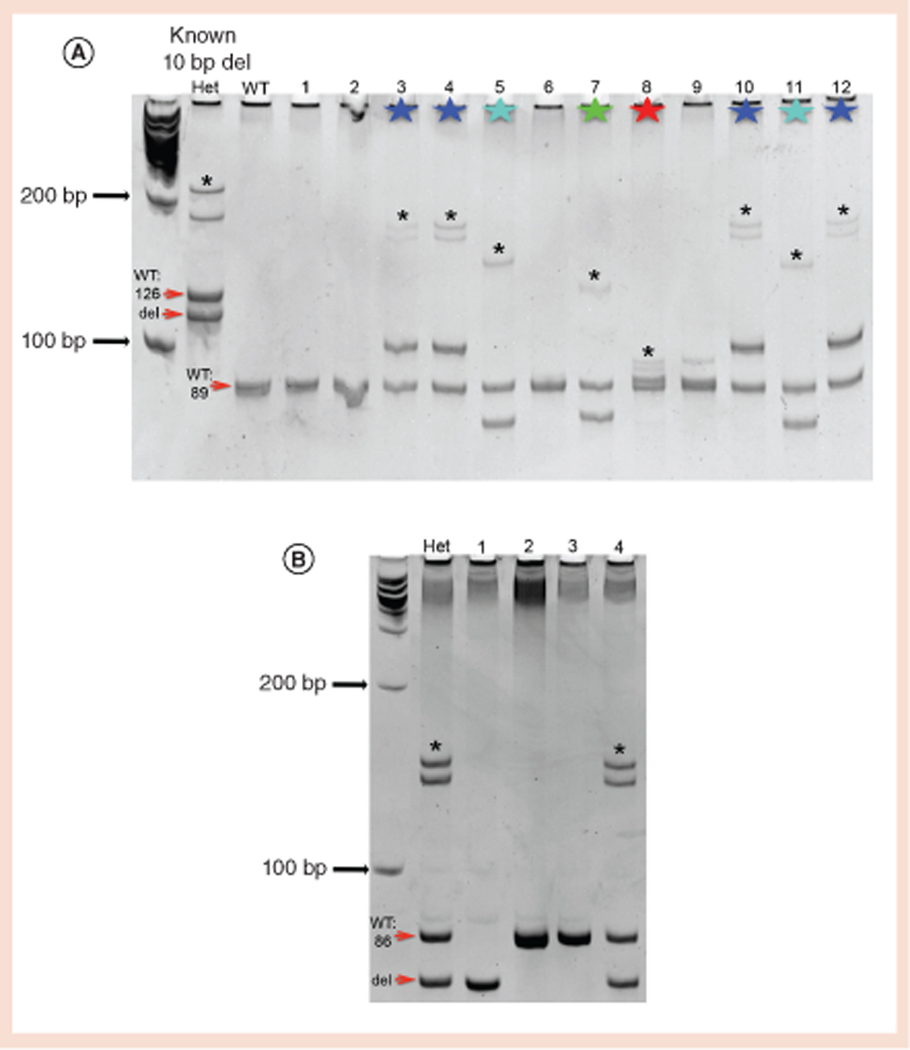
Representative ways in which neutral PAGE can be used to screen at the F1 and F2 generations. **(A)** PAGE results showing 12 individual embryos from an outcross in which the F0-injected founder transmits four different germline mutations in *gad1a* exon 5 at a frequency of ∼67%. The first lane is a known heterozygous zebrafish for a different allele that serves as a positive control. The starred samples were sequenced and determined to have the following types of mutations: embryo numbers 3, 4, 10, 12 have a 14-bp insertion; embryos 5 and 11 have a 10-bp deletion; embryo number 7 has a 9-bp deletion; embryo number 8 has a 2-bp insertion. **(B)** PAGE results from an incross of a line of fish (*gav2501*) that are heterozygous for a CRISPR-Cas9-induced 10-bp deletion at *gad2* exon 1. Fish number 1 is a homozygous mutant, fish numbers 2 and 3 are wild-type and fish number 4 is a heterozygous mutant. In both gels, the second pair of bands that are noted with an asterisk are heteroduplexes.

We typically detect mutations in 8–25% of our F0-injected zebrafish with a germline transmission rate between 25 and 67%. Using this approach, we have identified seven novel alleles at three different loci. Based upon the resolution power of the gels described above, we have tested amplicons ranging between 86 and 126 bp under these conditions and were consistently able to detect indels as small as 1 bp. Representative gels for an F0 outcross screening that led to the identification of four novel alleles as well as how this approach is used to genotype the F2 generation are shown in Figure 2.

The major benefits of this protocol compared with similar approaches are the improvements in sensitivity of the assay and the time– and cost–effectiveness. For instance, the genomic DNA extraction, PCR, and analysis of the polyacrylamide gel can be performed within 48 h, while the use of sequencing as a primary screening approach takes up to 3 days and is more expensive. Similarly, there is no enzymatic cleavage step like in T7E1 nor is there a need to heat the samples to form heteroduplexes between the PCR and PAGE [9,13,14]. Therefore, this approach saves at least one step that is required in other protocols and does not rely on analysis of mismatches that may also report SNPs.

This approach provides the ability to elucidate multiple pieces of mutagenesis information in a single experiment. Using PCR and PAGE as described here will show if an F0-injected animal is carrying a germline mutation at a frequency of at least 8% (if there is an indel in at least one of 12 putative F1 animals that are screened). This approach also shows the relative type of mutations present (insertions versus deletions) and how many types of mutations come from each F0-injected zebrafish (Figures 1 & 2, Panel A).

This approach is also well-suited as a genotyping method once known alleles are identified, since we can discriminate between wild-type, heterozygous and homozygous mutations under the same conditions as those for which we screen for novel mutations (Figure 2, Panel B). Finally, this protocol has been successfully used to detect CRISPR-Cas9-induced indels in other vertebrates in addition to zebrafish, including mice [Sumadra and Condie, Pers. Comm.], human iPSCs [Lauderdale, Unpublished data], and lizards (supplemental protocol).

## Supplementary Material

Supplementary Material
