# Supplementary material for: A PAGE screening approach for identifying CRISPR-Cas9-induced mutations in zebrafi sh
Source: Biotechniques. Author manuscript; Available in PMC 2022 Oct 19. (PMC9580339; doi:10.2144/btn-2018-0012)
Supplement: Supplementary Material [file NIHMS1575821-supplement-Supplementary_Material.docx]

Screening For CRISPR Mutants Using PAGE

Protocol For:

A polyacrylamide gel electrophoresis (PAGE) screening approach for identifying CRISPR-Cas9 induced mutations in zebrafish

**Ariel J. VanLeuven^1^ and James D. Lauderdale^1,2^**

*^1^Department of Cellular Biology, ^2^Faculty in the Neuroscience Division of the Biomedical and Health Sciences Institute (BHSI), University of Georgia, Athens, Georgia, 30602, USA*

*BioTechniques (February 2018)*

**LEGEND**

🢡***ATTENTION***

* ***HINT***

**🖐*REST***

**REAGENTS**

10X TBE (Bio-Rad Laboratories, Inc., Hercules, CA, USA) Cat. # 161-0733

Dream Taq DNA Polymerase (Thermo Fisher Scientific, Waltham, MA, USA) EP0702

10X Dream Taq Green Buffer (Thermo Fisher Scientific, Waltham, MA, USA)

100mM dNTPs (Thermo Fisher Scientific, Waltham, MA, USA) Cat. No 10297018

100 µM Oligonucleotide Primers (Integrated DNA Technologies, Inc., Redwood City, CA, USA)

100% Ethanol (Koptec, King of Prussia, PA, USA) CAS# 64-17-5

30% Acrylamide/Bis Solution 19:1 with 5% crosslinker (Bio-Rad Laboratories, Inc., Hercules, CA, USA) Cat. # 161-0154

TEMED N,N,N’,N’-Tetramethylethylenediamine (Research Organics, Inc., Cleveland, OH, USA) CAS# 110-18-9

Ammonium persulfate (Sigma-Aldrich, St. Louis, MO, USA) CAS: 7727-54-0

Quick-Load Purple 2-Log DNA Ladder (New England BioLabs, Inc., ) #N0550S

10 mg/mL Ethidium bromide (Bio-Rad Laboratories, Inc., Hercules, CA, USA) Cat. # 161-0433

**PROCEDURE**

Genomic DNA Extraction

1. Place a dechorinated zebrafish embryo (or a piece of zebrafish tail fin) into a 1.5 mL microfuge tube and remove the excess liquid.
2. Add 48 µL of DNA Extraction buffer & 2 µL of 10 mg/mL ProK in glycerol to each tube.
3. Incubate at 55°C in a water bath

**🖐*REST:*** *Leave the solution overnight in 55°C*

1. After the overnight incubation, centrifuge at 13,000 rpm for 10 minutes at room temperature.
2. Remove the pellet of debris and add 100 µL of 100% Ethanol to precipitate the genomic DNA.
3. Shake to mix and place tube(s) in -20°C for at least 30 minutes.

**🖐*REST:*** *Samples can be left/stored in* -*20°C as long as needed*

1. Centrifuge at 13,000 rpm for 10 minutes at room temperature.
2. Remove the supernatant and dry the pellet for ~1 minute.
3. Resuspend the genomic DNA in 21 µL of 1X TE Buffer

* ***HINT****: At this point, you can check the concentration of the genomic DNA spectrophotometrically and/or run a small aliquot on a 1.5% agarose gel, but for more experienced users, this step is not necessary*

**🖐*REST:*** *Either store the genomic DNA at 4°C or use immediately in PCR*

PCR

1. Assemble a PCR reaction to detect a CRISPR-induced mutation at a gene-specific target site as follows for a single reaction:

2.0 µL genomic DNA (this is ~100ng of DNA)

0.2 µL 20 µM Forward primer

0.2 µL 20 µM Reverse primer

2.0 µL 10X Dream Taq Green Buffer

0.5 µL 10mM dNTPs

0.1 µL Dream Taq (stock = 5U/μL)

15.0 µL MilliQ Water

**20uL**

* ***HINT****: Make a master mix for however many samples you have with all of these components except the genomic DNA, shake to mix, and aliquot 18* µL of the master mix to each tube.

1. Run the PCR in a Thermal Cycler.

🢡***ATTENTION:*** *Make sure to use the appropriate denaturing, annealing and extension temperatures which are dependent upon the type of Taq and the gene-specific primers. After the initial denaturation, use 30 cycles of denaturing, annealing and extension followed by a 2-minute 72°C extension.*

**🖐*REST:*** *The PCR product can be kept at 4°C or used immediately in PAGE.*

Polyacrylamide Gel Electrophoresis (PAGE)

🢡***ATTENTION:*** *Wear gloves at all times when handling the polyacrylamide gels as some of the reagents are neurotoxins.*

1. Use an upright electrophoresis setup (see equipment below for the model we use)

* ***HINT****:* This is an apparatus commonly used for SDS-PAGE and western blotting; glass plates and notched Alumina plates are both 10 x 8 cm; spacers are 10 cm x 1mm width T-shaped spacers

1. Rinse the glass plates, notched Alumina plates, spacers, and gel caster with water to remove any residual gel debris
2. Assemble the gel caster following the manufacturer protocol before preparing the gels
3. Make two 15% polyacrylamide gels (see recipe below)

* ***HINT****:* The acrylamide solution should not have less than 5% crosslinker.

* ***HINT****:* We do not place these solutions under a vacuum to de-gas/de-aerate before proceeding.

1. Once all ingredients are added, invert a couple times and quickly pour the mixture between the glass plates in the gel caster and fill it all the way to the top of the glass
2. Place a 15-well comb (1mm width) between the glass plates and into the liquid until the lips rest on the plates.
3. Allow the gels to polymerize/harden for ~45 minutes

* ***HINT****:* To ensure the gel is hardened, keep any remaining solution in the 15mL conical tube to monitor the polymerization process.

**🖐*REST:*** *Once made, the gel can be used immediately or stored in 1X TBE in 4°C*

1. Once the gel(s) have hardened, assemble the electrophoresis unit following the manufacturer protocol

* ***HINT****:* Do not re-use 1X TBE in the buffer chambers

1. When the PCR is complete, directly load 5 µL of each sample into individual wells on the gel; load 3 µL of DNA ladder as well
2. Set the voltage to 200V and run the gel for 2-2.5 hours

🢡***ATTENTION:*** *Do not run the gel longer than 3 hours at 200V or the buffer will overheat which results in poor gel resolution; image the gel immediately after the run*

Gel Analysis

1. Carefully remove the polyacrylamide gels from between the plates
2. Stain gels in ethidium bromide staining solution (see recipe below) for ~1 minute
3. De-stain the gels in cold tap water for ~1 minute before imaging
4. Place one gel at a time on the tray in the gel imager (see equipment below)
5. Adjust the camera and settings as prompted by the software to best visualize the entire gel
6. When positioned, press Capture to get a still image of the gel
7. Under the Image Tools icon, invert the data/display
8. Take a picture of the gel and save it as a tiff or jpg file
9. Repeat for the second gel

**RECIPES**

DNA Extraction Buffer (50mL)

1M Tris pH 8 500 µL 10 mM

0.5M EDTA 1 mL 10 mM
5M NaCl 2 mL 200 mM
20% SDS 1.25 mL 0.5%
MilliQ Water 45.25 mL

* ***HINT****:* If the SDS doesn’t go into solution, heat it at 37°C until it dissolves

10mg/mL ProK (200 µL)

20mg/mL ProK 100 µL 10 mg/mL
100% Glycerol 100 µL 50%

1X TE Buffer (50 mL)

1M Tris pH 8.2 500 µL 10 mM

0.5M EDTA 100 µL 1 mM

MilliQ Water 49.4 mL

10% w/v Ammonium persulfate (APS) (10 mL)

APS 1g

MilliQ Water up to 10 mL

5X TBE (10 mL)

10X TBE 5 mL 5X

MilliQ Water 5mL

1X TBE (1 L)

1X TBE 100 mL 1X

MilliQ Water 900 mL

20 µM Oligonucleotide Primers (50 µL)

100 µM primer 10 µL 20 µM

10mM Tris pH8 40 µL

10 mM dNTPs (50 µL)

100 mM dATPs 5 µL 10 mM

100 mM dCTPs 5 µL 10 mM

100 mM dGTPs 5 µL 10 mM

100 mM dTTPs 5 µL 10 mM

10mM Tris pH8 30 µL

15% Polyacrylamide Gel (12 mL, for 2 gels)

30% Acrylamide/Bis Solution 19:1 6 mL 15%

5X TBE 2.4 mL

MilliQ Water 3.6 mL

10% w/v APS 60 µL

TEMED 12 µL

Add the water, TBE and Polyacrylamide first (no particular order is necessary) followed by the APS and finally the TEMED in a 15mL conical tube. We do not de-gas the gel.

Ethidium Bromide Staining Solution

10 mg/mL Ethidium bromide 10 µL 0.5 µg/mL

Distilled Water up to 200 mL

**TROUBLESHOOTING**

Smeared or poorly resolved bands on gel

Poor DNA extraction; don’t use a crude DNA extraction approach (alkaline lysis), do a ProK extraction and ethanol precipitation.

Wavy bands on gel or poor gel resolution

Poor electrophoresis conditions; use fresh 1X TBE for each gel run or cool the buffer before use. Avoid running the gel longer than 3 hours.

Samples do not travel down the gel

Improper apparatus setup; make sure the seals are tight so that buffer doesn’t leak out during the run

**EQUIPMENT**

Vapo.Protect Thermal Cycler (Eppendorf, Hamburg, Germany, 6325ZJ104747)

SE 250 Mighty Small II Upright Electrophoresis Setup (Hoefer, Holliston, MA, USA, SE250-10A-1.0)

Molecular Imager® Gel Doc^™^ XR+ with Image Lab^™^ software (Bio-Rad Laboratories, Inc., Hercules, CA, USA, 721BR05583)
